# 3D foot and ankle radiographic measurement toolbox

**DOI:** 10.3389/fbioe.2026.1892094

**Published:** 2026-08-05

**Authors:** Andrew C. Peterson, Elana Renae Lapins, Melissa R. Requist, Karen M. Kruger, Amy L. Lenz

**Affiliations:** 1 Department of Mechanical Engineering, University of Utah, Salt Lake City, UT, United States; 2 Joint Department of Biomedical Engineering, Marquette University, Milwaukee, WI, United States; 3 Shriners Children’s, Chicago, IL, United States

**Keywords:** computational automatic toolbox, foot and ankle, foot deformities, radiographic measurements, three-dimensional imaging

## Abstract

Two-dimensional radiographic measurements remain the clinical standard for evaluating foot and ankle deformities, despite increasing use of three-dimensional weightbearing computed tomography (WBCT). While WBCT enables improved visualization of osseous relationships under static load, translation of familiar two-dimensional radiographic metrics into three-dimensional imaging environments has been limited by manual workflows and a lack of standardized computational frameworks. To address this gap, we developed the three-dimensional Foot and Ankle Radiographic Measurements (3D FARM) toolbox to automatically compute clinically relevant radiographic measurements from bone models. WBCT scans from 180 adult feet across six clinical groups (rectus, cavus, planus, Charcot-Marie-Tooth, progressive collapsing foot deformity, and tibiotalar/subtalar osteoarthritis) were analyzed. Fourteen bones per foot were segmented using both semi-automatic and manual methods. The 3D FARM toolbox automatically computes 20 commonly used radiographic measurements using anatomically defined coordinate systems. Agreement between segmentation methods was evaluated using intraclass correlation coefficients and mean bias. Measurements from reference manual segmentations were used to generate group-specific reference values. Agreement between semi-automatic and manual segmentations was excellent for 18 of 20 measurements, with the remaining measurements demonstrating good to moderate agreement. Mean bias across measurements was minimal. Group-specific reference values demonstrated distinct distributions across pathologies and aligned qualitatively with published radiographic measurements. The 3D FARM toolbox enables reproducible, automated extraction of clinically familiar radiographic measurements from WBCT data. Strong agreement with manual segmentation and establishment of adult pathology-specific reference values support its use as a standardized framework for three-dimensional foot and ankle deformity assessment.

## 1 Introduction

Three-dimensional (3D) imaging has transformed the evaluation of foot and ankle pathology by allowing accurate visualization of complex osseous relationships under weightbearing conditions (Lintz et al., 2018; Godoy-Santos et al., 2021; Godoy-Santos and Cesar, 2018). Weightbearing computed tomography (WBCT), in particular, enables detailed characterization of hindfoot alignment and deformity that is often obscured in two-dimensional (2D) radiographs (Kim et al., 2024a). Despite this growing accessibility, manual 2D radiographic measurements continue to be the clinical standard for quantifying deformity, surgical planning, and postoperative assessment (Bernasconi et al., 2024). Measurements such as Meary’s angle, calcaneal pitch, and hindfoot alignment angles are well established and readily interpretable by clinicians. However, the translation of these familiar parameters into 3D imaging environments has been limited by the need for time-intensive manual analysis and the lack of standardized computational frameworks (Pavani et al., 2022; Cai et al., 2024; Kruger et al., 2025).

Manual derivation of 2D-analog measurements from 3D models typically requires individualized bone segmentation, manual placement of anatomic landmarks, and linear definitions that vary across investigators (Krähenbühl et al., 2022; Sangoi et al., 2022; Bernasconi et al., 2021; Lintz et al., 2023). These processes are not only labor-intensive but also susceptible to user variability and inconsistent coordinate definitions, particularly in cases with complex or severe deformities such as cavovarus, planovalgus, or osteoarthritis (Knutson et al., 2023). As a result, quantitative comparisons between WBCT-based studies remain difficult, and the potential for large-scale, automated morphological assessment remains largely unrealized. In addition, pathology-specific descriptive values for 3D WBCT-derived radiographic-style measurements remain limited, making it difficult to interpret individual measurements or compare deformity patterns across clinical groups.

To address these limitations, we developed the 3D Foot and Ankle Radiographic Measurements (3D FARM) toolbox. This toolbox is currently a MATLAB-based framework that automatically calculates standard radiographic measurements from 3D bone models generated from WBCT scans. The toolbox integrates the Automatic Anatomical Foot and Ankle Coordinate Toolbox (AAFACT) to establish bone-specific coordinate system, ensuring consistent and anatomically grounded plane and axis definitions (Peterson et al., 2023). Using these coordinate systems, the toolbox computes 3D measurements designed to reflect clinical constructs commonly assessed using 2D radiographic measurements. The objectives of this study were twofold: 1) to evaluate the agreement between measurements derived from semi-automatic segmentation and manual segmentation, using intraclass correlation coefficients and mean bias to quantify measurement reproducibility; and 2) to establish group-specific descriptive values for all measurements across six pathological groups (rectus, cavus, planus, Charcot-Marie-Tooth (CMT), progressive collapsing foot deformity (PCFD), and tibiotalar/subtalar osteoarthritis (OA)) and qualitatively compare these to published 2D radiographic norms.

We hypothesized that (1) the 3D FARM toolbox would produce measurements on the semi-automatically derived segmentations in excellent agreement with measurements on the manual segmentations and (2) that group-specific 3D measurement trends would be qualitatively consistent with established 2D radiographic patterns of deformity. Together, these aims were designed to evaluate the reproducibility of 3D FARM measurements across segmentation workflows and to provide group-level descriptive values for adult foot and ankle pathologies.

## 2 Materials and methods

### 2.1 Participant screening and data preparation

**FIGURE 1:**
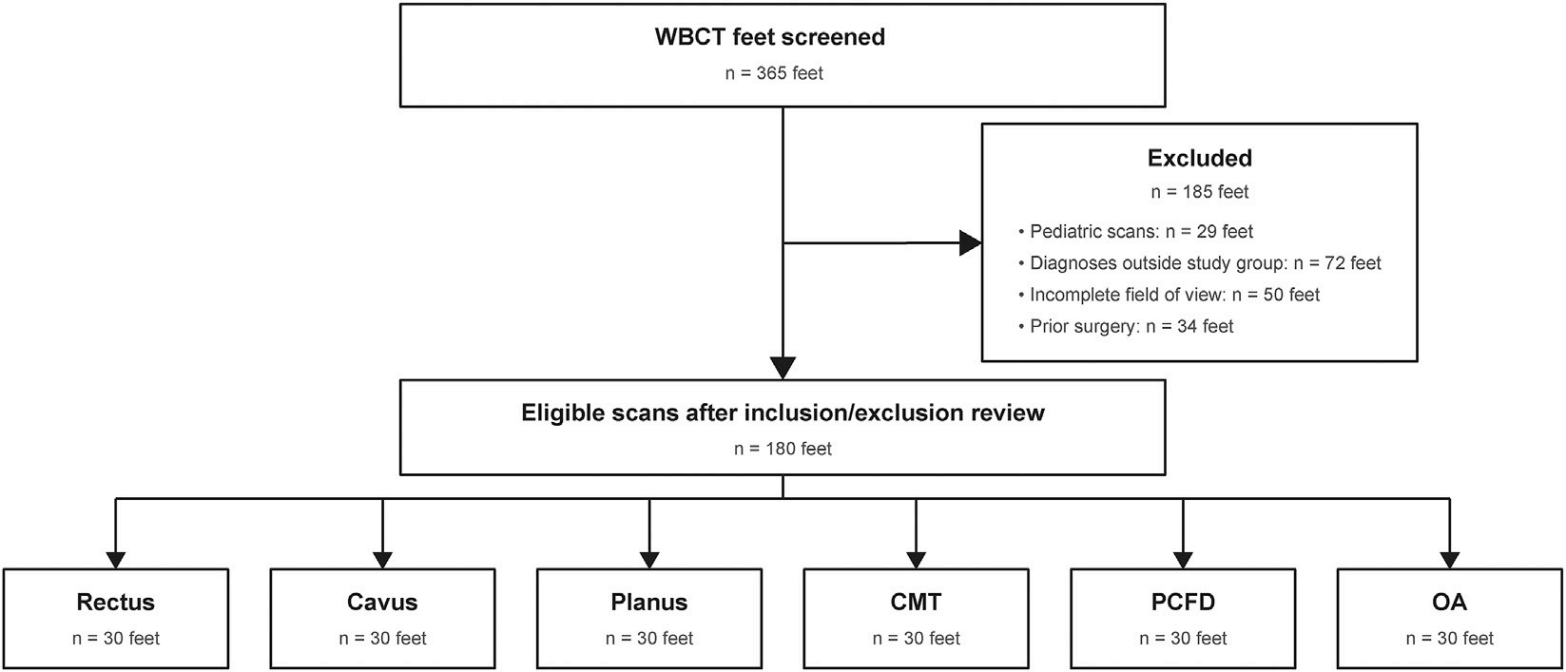
Participant selection flow diagram. Highlighting feet that were screened for inclusion, those excluded, and final analytic cohort number. The final cohort included recuts, cavus, planus, Charcot-Marie-Tooth disease (CMT), progressive collapsing foot deformity (PCFD), and tibiotalar/subtalar osteoarthritis (OA).

This retrospective imaging study analyzed WBCT scans from 180 adult individuals (48.8 ± 17.1 years; 83 female). Participants were categorized into six clinical groups based on a chart review, with 30 in each group: rectus, cavus, planus, CMT, PCFD, and tibiotalar/subtalar OA. These participants were assigned to groups based on retrospective review of clinic notes and radiology reports. Group assignment was based on the primary documented clinical diagnosis at the time of imaging rather than thresholds derived from 3D FARM measurements. No individuals included in this analysis had overlapping diagnoses. Rectus participants had no documented foot or ankle deformity diagnosis included in the pathology groups. Following Institutional Review Board (IRB) approval, WBCT scans meeting inclusion criteria were retrospectively identified. Scans were acquired using either a pedCAT (CurveBeam; 0.37 mm isotropic voxel size; n = 153) or HiRise (CurveBeam; 0.3 mm isotropic voxel size; n = 27). Inclusion criteria were adults with complete WBCT datasets and visible osseous anatomy from the tibia through the metatarsals. Exclusion criteria included poor image quality or prior foot and ankle surgeries. For bilateral WBCT scans, only one limb was analyzed. For individuals with a unilateral acute injury, the contralateral uninjured limb was analyzed. For all other individuals, the laterality was randomly selected (Figure 1).

For each individual, 14 bones (tibia, fibula, talus, calcaneus, navicular, cuboid, three cuneiforms, and five metatarsals) were segmented from each scan using two approaches: semi-automatic and manual semi-automatic segmentation was performed using commercially available clinical software (Paragon 28, DISIOR, Bonelogic Ortho Foot and Ankle, Helsinki, Finland), and manual segmentation was performed by trained engineers using an established and consistent workflow with manual reviews in Mimics (Materialise, Leuven, Belgium) and 3-matic (Materialise, Leuven, Belgium) for model smoothing. All segmentations were exported as STL files in preparation for input into the toolbox. Manual segmentations were used as the reference segmentation because they were generated by trained engineers using an established laboratory protocol, manually reviewed for anatomical accuracy, and processed consistently across all included bones.

### 2.2 Automatic measurement toolbox

**FIGURE 2:**
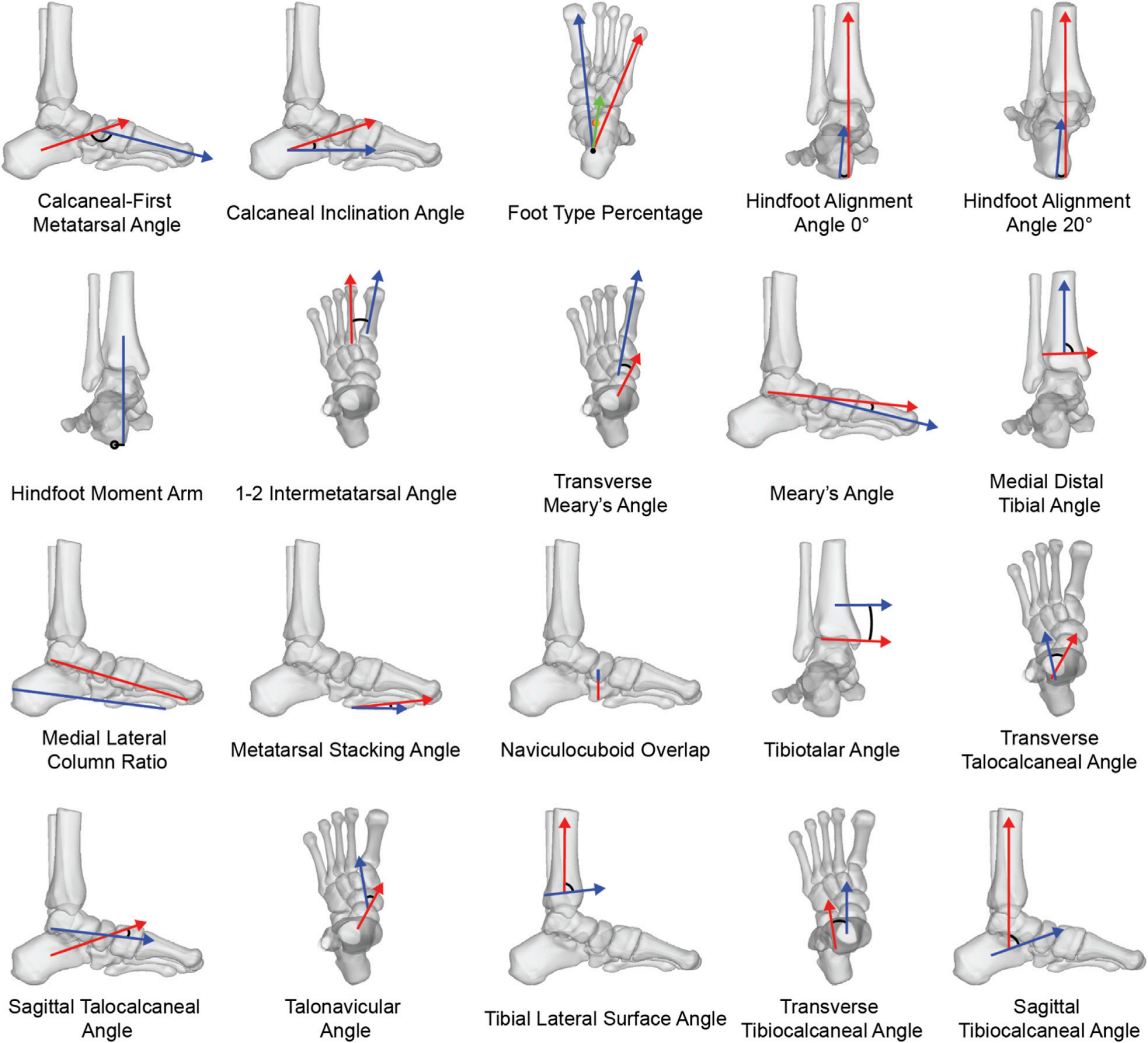
All measurements in the 3D Foot and Ankle Radiographic Measurements (3D FARM) toolbox. Arrows indicate defined anatomical axes with differing colors for ease of distinction. Planes and sign conventions are explained in Table 1.

A MATLAB (MathWorks, Natick, MA) toolbox, 3D FARM (https://github.com/Lenz-Lab/3DFARM), was developed to automatically compute clinically relevant radiographic measurements from 3D bone models (Peterson, 2026). The analyses described in this manuscript were performed using version 1.0 of the toolbox. The repository included licensing, instructions for use, dependencies, documentation, and an example dataset. The measurement workflow required approximately 1 min per foot, but could vary depending on computations specifications. The vectors used to calculate these measurements were based on the Automatic Anatomical Foot and Ankle Coordinate Toolbox (AAFACT) (Peterson et al., 2023), which establishes standardized, anatomically grounded axes. Using these vectors, 3D FARM calculates the projection of bone segmentations onto a simulated radiographic plane and computes angular or linear parameters mirroring common 2D clinical measures. The simulated radiographic planes were defined from a subject-specific global anatomical coordinate system derived from the AAFACT bone-specific coordinate systems. This global coordinate system combines anatomical axes from multiple bones to define consistent axes for each subject. The sagittal, coronal, and transverse projection planes were then defined from these global axes and were therefore patient-specific and automatically based rather than fixed to scanner coordinates or image orientation. Because the global coordinate system incorporates multiple bone-specific axes, the projection planes are designed to reduce sensitivity to any single irregularly aligned bone. The following measurements were calculated for each individual: calcaneal first metatarsal angle (C1M), calcaneal inclination angle (CIA), foot type percentage (FTP), hindfoot alignment angle (HAA) at 0° and 20° angles, hindfoot moment arm (HMA), first and second intermetatarsal angle, Meary’s angle in the transverse and sagittal planes, medial distal tibial angle (MDTA), medial-lateral column ratio (MLCR), metatarsal stacking angle, naviculocuboid overlap, talar tilt angle (TTA), talocalcaneal angle (TCA) in the transverse and sagittal planes, talonavicular angle (TNA), tibial lateral surface angle (TLSA), and tibiocalcaneal angle (TiCA) in the transverse and sagittal planes (Table 1; Figure 2). FTP captures a related construct to Foot and Ankle Offset (Lintz et al., 2023), but should be interpreted as a distinct 3D FARM measurement. All measurements were automatically calculated in the toolbox.

**TABLE 1:** Current 3DFARM measurement definitions.

Measurement	Bones/Axes used	Plane	Description	Units
C1M	AP 1st Metatarsal vs. AP Calcaneus	Sagittal	↑ value = ↓ arch height	Degrees
CIA	AP Global vs. AP Calcaneus	Sagittal	↑ value = ↑ inclination	Degrees
FTP	AP Calcaneus, AP 1st Metatarsal, AP 5th Metatarsal, and Superior Talar Point	Transverse	↑ value = ↑ valgus	Percent
HAA 0°	SI Calcaneus vs. SI Tibia	Coronal	↑ value = ↑ valgus	Degrees
HAA 20°	SI Calcaneus vs. SI Tibia	Coronal with 20° Superior Rotation	↑ value = ↑ valgus	Degrees
HMA	SI Tibia vs. Inferior Calcaneal Point	Coronal	↑ value = ↑ lateral shift	Millimeters
1-2IMA	AP 1st Metatarsal vs. AP 2nd Metatarsal	Transverse	↑ value = ↑ metatarsal splay	Degrees
Transverse Meary’s angle	AP 1st Metatarsal vs. AP Talus	Transverse	↑ value = ↑ valgus	Degrees
Sagittal Meary’s angle	AP 1st Metatarsal vs. AP Talus	Sagittal	↑ value = ↑ arch height	Degrees
MDTA	SI Tibia vs. ML Tibial Plafond	Coronal	↑ value = ↑ lateral tilt	Degrees
MLCR	Posterior Talus to Anterior 1st Metatarsal vs. Posterior Calcaneus to Anterior 5th Metatarsal	N/A	↑ value = ↑ medial column dominance	N/A
Metatarsal Stacking angle	AP 5th Metatarsal vs. Anterior 1st Metatarsal	Sagittal	↑ value = ↓ arch height	Degrees
Naviculocuboid Overlap	Cuboid Origin to Inferior Cuboid vs. Cuboid Origin to Inferior Navicular	Sagittal	↑ value = ↓ arch height	N/A
TTA	ML Tibia vs. ML Talus	Coronal	↑ value = ↑ varus	Degrees
Transverse TCA	AP Calcaneus vs. AP Talus	Transverse	↑ value = ↑ internal rotation	Degrees
Sagittal TCA	AP Talus vs. AP Calcaneus	Sagittal	↑ value = ↑ plantarflexion	Degrees
TNA	AP Talus vs. AP Navicular	Transverse	↑ value = ↑ internal rotation	Degrees
TLSA	AP Tibial Plafond vs. SI Tibia	Sagittal	↑ value = ↑ posterior tilt	Degrees
Transverse TiCA	AP Tibia vs. AP Calcaneus	Transverse	↑ value = ↑ valgus	Degrees
Sagittal TiCA	AP Calcaneus vs. AP Tibia	Sagittal	↑ value = ↑ plantarflexion	Degrees

Abbreviations: AP, anterior-posterior; SI, superior-inferior; ML, medial-lateral; C1M, calcaneal first metatarsal angle; CIA, calcaneal inclination angle; FTP, Foot Type Percentage; hindfoot alignment angle (HAA) at 0° and 20° angles, HMA, hindfoot moment arm; first and second intermetatarsal angle; medial distal tibial angle (MDTA), 1-2IMA, MLCR, medial-lateral column ratio; TTA, talar tilt angle; talocalcaneal angle (TCA) in the transverse and sagittal planes, TNA, talonavicular angle; TLSA, tibial lateral surface angle; and tibiocalcaneal angle (TiCA) in the transverse and sagittal planes.

### 2.3 Statistical analysis

To evaluate measurement agreement between semi-automatic (DISIOR) and manual (Mimics) segmentations, all measurements were compared across the full cohort and within groups. Intraclass correlation coefficient (ICC) was used to quantify absolute agreement for each measurement, with excellent ≥0.90, good ≥0.75, moderate ≥0.50, and poor <0.50 (Koo and Li, 2016). Bland-Altman analysis was also performed for each measurement across the full cohort. For each participant, the measurement difference was calculated as DISIOR minus Mimics, and the paired measurement mean was calculated as the average of the DISIOR- and Mimics-derived values. Mean bias and limits of agreement (LOA), mean absolute error (MAE), and root mean square error (RMSE) were calculated to further describe measurement agreement. All calculations were performed with a two-way random effects, absolute-agreement ICC.

For each foot type or pathology group, descriptive statistics were calculated from Mimics-derived measurements to establish group-level descriptive values. For each measurement, the mean, standard deviation, and 95% confidence intervals (CI) of the mean were computed. These values were compared qualitatively with published 2D radiographic norms to assess alignment with expected deformity patterns.

To evaluate whether scanner type influenced measurement agreement, a scanner-based sensitivity analysis was performed. Agreement metrics were calculated separately for pedCAT and HiRise scans, and ICC values were compared between workflows within each group.

## 3 Results

### 3.1 Segmentation-based agreement analysis

**FIGURE 3:**
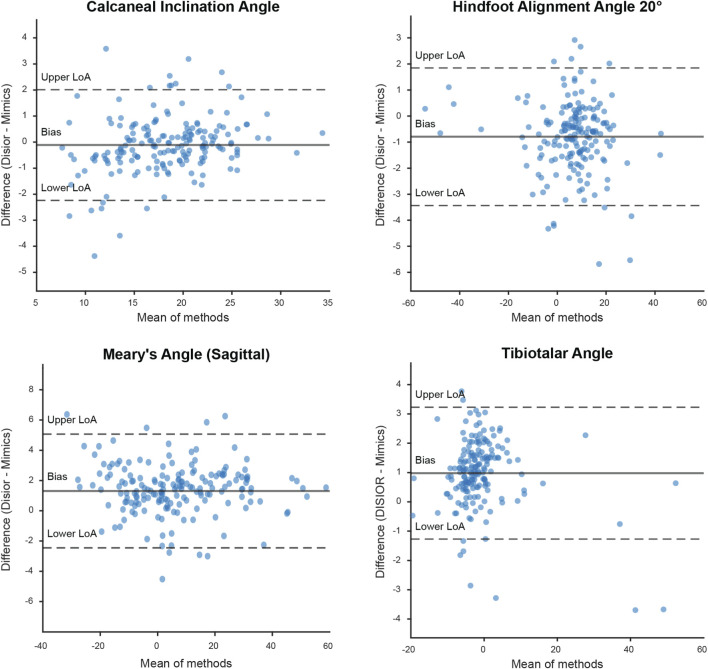
Bland-Altman plots for four commonly used measurements as a supplement figure for Table 2.

All radiographic measurements were successfully computed for all 180 adult feet using both DISIOR and Mimics segmentations. Overall agreement between segmentation methods was high across all measurements (Table 2). ICC values demonstrated excellent agreement in 18 of 20 measurements, with TLSA and MDTA exhibiting good or moderate agreement. Bland-Altman analysis demonstrated small mean bias values across measurements, with bias values ranging from −1.56–2.67° or millimeters, depending on the metric (Table 2). Representative Bland-Altman plots for four commonly used measurements are shown in Figure 3, with statistics for all measurements reported in Table 2.

**TABLE 2:** Radiographic measurements with overall intraclass correlation coefficient (ICC) with 95% confidence intervals (CI), mean bias, mean absolute error (MAE), root mean square error (RMSE), and limits of agreement (LOA).

Measurement	ICC (95% CI)	Bias	MAE	RMSE	LOA
C1M	1.00 (1.00–1.00)	−0.37°	0.99	1.35	−2.92–2.18
CIA	0.98 (0.97–0.98)	−0.11°	0.78	1.09	−2.24–2.01
FTP	0.98 (0.96–0.99)	0.45%	1.06	1.53	−2.41–3.31
HAA 0°	0.99 (0.99–0.99)	−0.89°	1.30	1.74	−3.83–2.06
HAA 20°	0.99 (0.99–1.00)	−0.79°	1.17	1.56	−3.44–1.85
HMA	0.99 (0.98–0.99)	−1.56 mm	1.96	2.53	−5.45–2.33
1-2IMA	0.96 (0.93–0.98)	−0.16°	0.58	0.95	−1.99–1.68
Transverse Meary’s angle	0.99 (0.99–1.00)	0.29°	1.46	2.03	−3.65–4.23
Sagittal Meary’s angle	0.99 (0.99–0.99)	1.31°	1.87	2.32	−2.45–5.07
MDTA	0.73 (0.61–0.82)	−1.22°	1.55	2.13	−4.64–2.20
MLCR	0.94 (0.90–0.97)	0.00	0.01	0.02	−0.05–0.04
Metatarsal Stacking angle	0.93 (0.85–0.97)	−0.30°	1.34	2.45	−5.06–4.46
Naviculocuboid Overlap	0.97 (0.96–0.98)	2.67	3.66	5.67	−7.13–12.47
TTA	0.99 (0.97–0.99)	0.98°	1.26	1.51	−1.27–3.23
Transverse TCA	0.96 (0.94–0.97)	−0.81°	1.63	2.30	−5.03–3.40
Sagittal TCA	0.90 (0.86–0.93)	−0.94°	1.63	2.15	−4.79–2.85
TNA	0.99 (0.99–0.99)	0.09°	2.02	2.61	−5.02–5.20
TLSA	0.85 (0.77–0.91)	−0.66°	1.37	2.05	−4.46–2.05
Transverse TiCA	0.96 (0.94–0.97)	0.58°	1.49	2.09	−3.36–4.51
Sagittal TiCA	0.99 (0.99–0.99)	−0.12°	0.78	1.12	−2.31–2.07

Abbreviations: C1M, calcaneal first metatarsal angle; CIA, calcaneal inclination angle; FTP, Foot Type Percentage; hindfoot alignment angle (HAA) at 0° and 20° angles, HMA, hindfoot moment arm; 1-2IMA, first and second intermetatarsal angle; MDTA, medial distal tibial angle; MLCR, medial-lateral column ratio; TTA, talar tilt angle; talocalcaneal angle (TCA) in the transverse and sagittal planes, TNA, talonavicular angle; TLSA, tibial lateral surface angle; and tibiocalcaneal angle (TiCA) in the transverse and sagittal planes.

Agreement patterns were consistent across pathology groups. Per-group ICC and bias values demonstrated similar trends to the overall cohort, with the majority of comparisons having excellent agreement and sub-degree mean bias values. Also similar to the overall comparison, MDTA and TLSA had some of the worst ICC values among each group.

Scanner-specific sensitivity analysis demonstrated similar agreement patterns between machines for most measurements. Scanner-specific ICC values differed by less than 0.1 for all measurements except MDTA and TLSA. These two measurements had drastically better ICC values from scans in the pedCAT (∼0.85) compared to the HiRise (∼0.35).

### 3.2 Clinical reference data

The measurements from the Mimics segmentations were used to generate group-specific reference values for all radiographic parameters. For each pathology group, the mean, standard deviation, and 95% CI of the mean were calculated (Table 3).

**TABLE 3:** Radiographic measurements with group specific mean and standard deviation (SD) with a 95% confidence interval and commonly used literature ranges.

Group	Measurement	Mean ± SD	95% CI	Literature range*
Rectus	C1M	147.0° ± 6.1°	144.7°–149.3°	​
Rectus	CIA	18.5° ± 3.0°	17.3°–19.6°	11.0°–20.9° (Sangoi et al., 2022)
Rectus	FTP	3.1% ± 2.3%	2.2%–4.0%	−0.7% – 2.8% (Bernasconi et al., 2021; Pires et al., 2024; Bernasconi et al., 2020)
Rectus	HAA 0°	9.2° ± 3.5°	7.9°–10.5°	​
Rectus	HAA 20°	8.5° ± 3.2°	7.3°–9.7°	4.1°–18° (Pires et al., 2024; Buck et al., 2011)
Rectus	HMA (mm)	14.8 ± 4.7	13.0–16.5	​
Rectus	1-2IMA	12.1° ± 3.3°	10.9°–13.3°	​
Rectus	Trans. Meary’s angle	7.8° ± 5.4°	5.8°–9.8°	​
Rectus	Sagittal Meary’s angle	4.0° ± 4.8°	2.2°–5.8°	−4.6° – 6.0° (Sangoi et al., 2022; Bernasconi et al., 2021)
Rectus	MDTA	90.6° ± 2.9°	89.5°–91.6°	​
Rectus	MLCR	1.0 ± 0.0	1.0–1.0	​
Rectus	Metatarsal Stacking angle	6.1° ± 2.7°	5.1°–7.1°	​
Rectus	Naviculocuboid Overlap	38.6 ± 13.4	33.6–43.6	​
Rectus	TTA	−3.5° ± 2.1°	−4.3° to −2.7°	−1.0° – 0.8° (Ranjit et al., 2023; Krähenbühl et al., 2022)
Rectus	Trans. TCA	24.3° ± 5.3°	22.3°–26.2°	26.1°–37.1° (Krähenbühl et al., 2022)
Rectus	Sagittal TCA	29.0° ± 4.0°	27.6°–30.5°	​
Rectus	TNA	28.6° ± 7.9°	25.7°–31.6°	29.4°–33.7° (Krähenbühl et al., 2022; Ranjit et al., 2023)
Rectus	TLSA	83.8° ± 2.5°	82.9°–84.8°	​
Rectus	Trans. TiCA	2.2° ± 5.3°	0.2°–4.1°	​
Rectus	Sagittal TiCA	70.9° ± 4.9°	69.1°–72.8°	​
Cavus	C1M	131.6° ± 9.4°	128.1°–135.1°	​
Cavus	CIA	21.6° ± 3.6°	20.3°–23.0°	18.6°–30.9° (Bernasconi et al., 2021; Fu et al., 2025; Togei et al., 2022; Chen et al., 2023)
Cavus	FTP	−3.0% ± 5.1%	−4.9% to −1.1%	−12.1% to −3.2% (Bernasconi et al., 2021; Bernasconi et al., 2020; Pires et al., 2024)
Cavus	HAA 0°	2.9° ± 6.4°	0.4°–5.3°	​
Cavus	HAA 20°	2.7° ± 6.0°	0.4°–4.9°	−20.5° – 1.6° (Pires et al., 2024; Togei et al., 2022; Chen et al., 2023)
Cavus	HMA (mm)	7.9 ± 7.2	5.2–10.6	​
Cavus	1-2IMA	9.4° ± 3.0°	8.3°–10.5°	​
Cavus	Trans. Meary’s angle	−8.8° ± 18.1°	−15.5° to −2.0°	​
Cavus	Sagittal Meary’s angle	21.7° ± 9.4°	18.2°–25.2°	6.0°–19.3° (Bernasconi et al., 2021; Fu et al., 2025; Togei et al., 2022; Zingas and King, 2025; Chen et al., 2023)
Cavus	MDTA	89.7° ± 2.5°	88.8°–90.6°	​
Cavus	MLCR	1.0 ± 0.1	0.9–1.0	​
Cavus	Metatarsal Stacking angle	4.9° ± 3.8°	3.5°–6.4°	​
Cavus	Naviculocuboid Overlap	14.5 ± 20.1	7.0–22.0	​
Cavus	TTA	−1.7° ± 3.5°	−3.0° to −0.4°	0.4°–9.2° (Tracey et al., 2019)
Cavus	Trans. TCA	19.2° ± 5.8°	17.0°–21.3°	16.0°–32.0° (Zingas and King, 2025)
Cavus	Sagittal TCA	26.7° ± 5.0°	24.8°–28.6°	​
Cavus	TNA	10.5° ± 14.8°	5.0°–16.0°	−23.3° – 15.0° (Togei et al., 2022; Zingas and King, 2025)
Cavus	TLSA	84.9° ± 2.8°	83.9°–86.0°	​
Cavus	Trans. TiCA	1.3° ± 6.0°	−0.9° – 3.6°	​
Cavus	Sagittal TiCA	68.8° ± 7.4°	66.0°–71.6°	​
Planus	C1M	154.8° ± 7.6°	152.0°–157.7°	​
Planus	CIA	16.6° ± 3.8°	15.2°–18.0°	9.6°–20° (Matsumoto et al., 2023; Ghaznavi et al., 2022; Alsaidi and Moria, 2023)
Planus	FTP	5.5% ± 2.0%	4.8%–6.3%	​
Planus	HAA 0°	14.2° ± 6.0°	12.0°–16.4°	​
Planus	HAA 20°	12.6° ± 5.0°	10.7°–14.4°	11.0°–18.0° (Stichnoth et al., 2025)
Planus	HMA (mm)	19.6 ± 6.9	17.0–22.2	​
Planus	1-2IMA	12.3° ± 2.3°	11.5°–13.2°	​
Planus	Trans. Meary’s angle	16.5° ± 7.7°	13.7°–19.4°	​
Planus	Sagittal Meary’s angle	−6.2° ± 5.9°	−8.4° to −3.9°	−29.1° to −4.0° (Matsumoto et al., 2023; Stichnoth et al., 2025)
Planus	MDTA	90.2° ± 2.0°	89.4°–91.0°	​
Planus	MLCR	1.0 ± 0.0	1.0–1.0	​
Planus	Metatarsal Stacking angle	7.7° ± 3.2°	6.5°–8.9°	​
Planus	Naviculocuboid Overlap	56.3 ± 10.4	52.4–60.2	​
Planus	TTA	−4.0° ± 3.1°	−5.2° to −2.9°	​
Planus	Trans. TCA	26.9° ± 3.5°	25.6°–28.2°	29.5°–41.5° (Ghaznavi et al., 2022)
Planus	Sagittal TCA	31.3° ± 3.8°	29.9°–32.8°	​
Planus	TNA	41.8° ± 7.2°	39.1°–44.5°	13.9°–40.1° (Ghaznavi et al., 2022; Matsumoto et al., 2023; Louie et al., 2014)
Planus	TLSA	84.1° ± 2.6°	83.1°–85.1°	​
Planus	Trans. TiCA	1.9° ± 7.1°	−0.8° – 4.6°	​
Planus	Sagittal TiCA	75.8° ± 7.1°	73.2°–78.5°	​
CMT	C1M	129.0° ± 14.3°	123.7°–134.4°	​
CMT	CIA	22.2° ± 5.0°	20.3°–24.0°	17.3°–36.0° (Chen et al., 2025; Sangoi et al., 2022; Faldini et al., 2015; Song et al., 2024; Bernasconi et al., 2021)
CMT	FTP	−6.7% ± 8.3%	−9.7% to −3.6%	−17.7% to −10.6% (Bernasconi et al., 2021; Bernasconi et al., 2020)
CMT	HAA 0°	−6.4° ± 21.1°	−14.3° – 1.4°	​
CMT	HAA 20°	−5.8° ± 19.5°	−13.1° – 1.4°	−7.8° – 29.8° (Song et al., 2024)
CMT	HMA (mm)	−2.7 ± 23.4	−11.4–6.1	​
CMT	1-2IMA	9.4° ± 2.5°	8.4°–10.3°	​
CMT	Trans. Meary’s angle	−10.7° ± 19.5°	−18° to −3.4°	​
CMT	Sagittal Meary’s angle	25.7° ± 15.4°	20.0°–31.5°	11.3°–26° (Chen et al., 2025; Sangoi et al., 2022; Faldini et al., 2015; Song et al., 2024; Bernasconi et al., 2021)
CMT	MDTA	91.3° ± 3.0°	90.1°–92.4°	​
CMT	MLCR	0.9 ± 0.1	0.9–0.9	​
CMT	Metatarsal Stacking angle	2.7° ± 12.1°	−1.8° – 7.2°	​
CMT	Naviculocuboid Overlap	10.3 ± 24.4	1.2–19.4	​
CMT	TTA	5.7° ± 17.4°	−0.8° – 12.2°	1.3°–3.7° (Ranjit et al., 2023; Song et al., 2024)
CMT	Trans. TCA	21.7° ± 10.2°	17.9°–25.5°	18.0°–22.0° (Chen et al., 2025)
CMT	Sagittal TCA	25.2° ± 4.2°	23.6°–26.8°	​
CMT	TNA	8.4° ± 22.1°	0.2°–16.7°	3.6°–6.4° (Song et al., 2024; Ranjit et al., 2023)
CMT	TLSA	87.9° ± 3.5°	86.6°–89.2°	​
CMT	Trans. TiCA	−1.4° ± 9.9°	−5.1° – 2.3°	​
CMT	Sagittal TiCA	67.1° ± 7.3°	64.4°–69.8°	​
PCFD	C1M	165.0° ± 7.7°	162.1°–167.9°	​
PCFD	CIA	13.2° ± 3.2°	12.0°–14.4°	0.8°–17.1° (Kim et al., 2025; Kim et al., 2024b; Flury et al., 2022; Osman et al., 2021)
PCFD	FTP	8.7% ± 4.3%	7.1%–10.4%	6.3%–14.9% (Bernasconi et al., 2025; Mansur et al., 2023)
PCFD	HAA 0°	18.1° ± 12.2°	13.6°–22.7°	​
PCFD	HAA 20°	15.1° ± 9.8°	11.5°–18.7°	6.0°–27.1° (Kim et al., 2025; Kim et al., 2024b; Bernasconi et al., 2025)
PCFD	HMA (mm)	19.8 ± 9.7	16.2–23.5	​
PCFD	1-2IMA	12.5° ± 4.7°	10.8°–14.3°	​
PCFD	Trans. Meary’s angle	33.0° ± 15.7°	27.1°–38.8°	​
PCFD	Sagittal Meary’s angle	−16.2° ± 7.5°	−19.0° to −13.4°	−46.0° to −10.9° (Kim et al., 2025; Bernasconi et al., 2025; Kim et al., 2024b; Flury et al., 2022; Osman et al., 2021)
PCFD	MDTA	91.2° ± 2.1°	90.5°–92.0°	​
PCFD	MLCR	1.0 ± 0.0	1.0–1.0	​
PCFD	Metatarsal Stacking angle	6.8° ± 5.2°	4.9°–8.8°	​
PCFD	Naviculocuboid Overlap	57.5 ± 10.0	53.8–61.3	​
PCFD	TTA	−4.6° ± 4.6°	−6.4° to −2.9°	−6.0° – 17.1° (Kim et al., 2025; Bernasconi et al., 2025; Kim et al., 2024b; Mansur et al., 2023; Krähenbühl et al., 2022)
PCFD	Trans. TCA	34.4° ± 7.4°	31.7°–37.2°	28.0°–30.5° (Kim et al., 2025; Krähenbühl et al., 2022; Osman et al., 2021)
PCFD	Sagittal TCA	31.2° ± 4.0°	29.7°–32.7°	​
PCFD	TNA	51.8° ± 13.3°	46.9°–56.8°	22.5°–57.4° (Kim et al., 2025; Bernasconi et al., 2025; Mansur et al., 2023; Krähenbühl et al., 2022; Osman et al., 2021)
PCFD	TLSA	85.4° ± 2.6°	84.4°–86.3°	​
PCFD	Trans. TiCA	0.0° ± 7.7°	−2.9° – 2.9°	​
PCFD	Sagittal TiCA	83.1° ± 7.6°	80.3°–86.0°	​
OA	C1M	147.0° ± 10.9°	143.0°–151.1°	​
OA	CIA	18.0° ± 3.8°	16.6°–19.4°	−0.6° – 19.4° (Fujimaki et al., 2023; Lithgow et al., 2024)
OA	FTP	2.1% ± 6.1%	−0.2% – 4.3%	−7.4% – 7.7% (VandeLune et al., 2022; Zhang et al., 2023)
OA	HAA 0°	9.3° ± 15.1°	3.7°–15.0°	​
OA	HAA 20°	8.2° ± 13.5°	3.1°–13.2°	6.8°–29° (Choi et al., 2025; Kvarda et al., 2023; Perisano et al., 2023)
OA	HMA (mm)	13.5 ± 16.2	7.5–19.6	​
OA	1-2IMA	9.9° ± 2.8°	8.9°–11.0°	​
OA	Trans. Meary’s angle	9.7° ± 13.0°	4.8°–14.5°	​
OA	Sagittal Meary’s angle	4.9° ± 12.4°	0.3°–9.6°	−5.4° – 5.8° (Kyung et al., 2025)
OA	MDTA	89.5° ± 2.6°	88.5°–90.5°	​
OA	MLCR	1.0 ± 0.01	1.0–1.0	​
OA	Metatarsal Stacking angle	6.1° ± 6.6°	3.7°–8.6°	​
OA	Naviculocuboid Overlap	34.3 ± 18.1	27.6–41.1	​
OA	TTA	−1.7° ± 7.8°	−4.6° – 1.2°	−12.4° – 13.5° (Choi et al., 2025; Kyung et al., 2025; Cao et al., 2025; Gong et al., 2024; Fujimaki et al., 2023)
OA	Trans. TCA	26.3° ± 7.4°	23.5°–29.0°	28.5°–46.7° (Kvarda et al., 2023)
OA	Sagittal TCA	28.0° ± 4.7°	26.3°–29.8°	​
OA	TNA	27.2° ± 16.0°	21.2°–33.2°	7.2°–18.6° (Efrima et al., 2024; Wang et al., 2025)
OA	TLSA	88.4° ± 4.7°	86.6°–90.1°	​
OA	Trans. TiCA	1.2° ± 8.1°	−1.8° – 4.3°	​
OA	Sagittal TiCA	75.0° ± 5.5°	73.0°–77.1°	​

Abbreviations: C1M, calcaneal first metatarsal angle; CIA, calcaneal inclination angle; FTP, Foot Type Percentage; hindfoot alignment angle (HAA) at 0° and 20° angles, HMA, hindfoot moment arm; 1-2IMA, first and second intermetatarsal angle; MDTA, medial distal tibial angle; MLCR, medial-lateral column ratio; TTA, talar tilt angle; talocalcaneal angle (TCA) in the transverse and sagittal planes, TNA, talonavicular angle; TLSA, tibial lateral surface angle; and tibiocalcaneal angle (TiCA) in the transverse and sagittal planes.

* Published literature ranges are included for qualitative context only. These ranges were derived from studies with differences in cohort composition, imaging modality, projection plane, measurement definitions, and disease severity.

## 4 Discussion

This paper introduces an open-source toolbox to automatically compute clinically relevant radiographic measurements from 3D WBCT bone models. The findings of this study were twofold. First, the 3D FARM toolbox generally demonstrated excellent agreement between measurements on semi-automatic segmentations compared to measurements on manual segmentations, with some measurement outliers. Second, the toolbox enabled a generation of comprehensive, pathology-specific 3D reference values derived from segmented WBCT scans, providing a standardized dataset of clinically familiar radiographic measurements across multiple deformity types. Together, these findings support the use of the 3D FARM toolbox as a reliable and scalable method for extracting conventional radiographic measurements from 3D data.

The primary agreement objective of this study was to determine whether measurements derived from semi-automatic segmentations were comparable to those obtained from manual segmentations. Overall, agreement between segmentation types was excellent with small mean bias values. The two measurements not achieving excellent agreement, MDTA and TLSA, are solely dependent on the tibia and the length of the segmented tibia may vary between segmentation approaches and therefore can affect the measurement output. However, the mean bias for these measurements was −1.22° and −0.66°, respectively, suggesting limited systematic offset between segmentation methods. Group specific analyses showed a similar pattern, with greater variability in agreement for MDTA and TLSA than for the other measurements. The scanner-based sensitivity analysis further supported this interpretation, as the largest scanner-related differences were observed for MDTA and TLSA. Together, these findings suggest that most 3D FARM measurements are robust across segmentation workflows and scanner types, while tibia-dependent measurements may be more sensitive to differences in field of view and segmented tibial length (Muhlrad et al., 2022).

The second objective of this study was to provide group-level descriptive values for commonly used radiographic measurements derived from 3D imaging which are lacking in current literature. The group-specific measurement trends observed in this study were generally consistent with established radiographic descriptions from the literature (Table 3), although some values differed from published 2D ranges. It is important to view these values as preliminary descriptive benchmarks and not diagnostic reference intervals. Absolute numeric equivalence between 2D radiographs and 3D WBCT-derived measurements is not expected due to differences in imaging modality, projection plane definition, measurement implementation, and cohort composition. However, the directionality and relative magnitude of group differences suggest that these 3D measurements may reflect related deformity constructs traditionally assessed using 2D imaging. Specific cutoff values used in diagnostics may therefore need to be adapted for use with 3D imaging (Kruger et al., 2025).

Automated extraction of familiar radiographic measurements from WBCT data may facilitate broader clinical adoption of 3D imaging by reducing reliance on manual measurement workflows. In the present study, 3D FARM computed all 20 measurements in approximately 1 min per foot. Although formal timed comparison with manual measurement workflows was not performed, this runtime supports the potential scalability of the automated workflow. Such tools have the potential to improve efficiency, reduce variability, and support more objective deformity characterization in both clinical and research settings. The reference data provided in the study may serve as a benchmark for evaluating deformity severity, surgical correction, or disease progression using WBCT. However, these values should be interpreted in the context of known differences between 2D and 3D measurement modalities.

Comparable commercial and semi-automated 3D WBCT analysis tools have been developed foot and ankle assessment, which have helped advance the use of WBCT 3D measurements. The goal of 3D FARM is to add a layer of transparency and reproducible framework for calculating clinically familiar radiographic-style measurements from segmented 3D bone models. A key distinction is that the coordinate systems, definitions, and source code are publicly available and version controlled, allowing investigators to inspect and reproduce the measurement workflow. In addition, the present study evaluates multiple measurements across various clinical groups for group-level descriptive values and includes a sensitivity analysis, positioning 3D FARM as a complementary framework for standardizing and studying 3D measurements across diverse foot and ankle pathologies.

This study has several limitations. First, this was a retrospective imaging study, and participants were selected from available WBCT scans meeting the study inclusion criteria, which may have inherent selection bias. Second, clinical groups were assigned based on chart review and documented clinical diagnosis, which may introduce group heterogeneity related to disease severity and clinical indication for imaging. Third, although group-specific descriptive values were compared qualitatively with published 2D radiographic ranges, direct agreement was not evaluated. Fourth, this study evaluated measurement agreement between segmentation workflows, but did not include validation against a ground truth, primarily because that does not exist reliably. Fifth, manual segmentation was treated as the reference standard, despite inherent observer variability. Additionally, smoothing was applied to the manual segmentation workflow but not to the DISIOR segmentations. Although the average surface displacement from smoothing was small relative to the scan voxel size, smoothing may still have a minor effect of surface geometry and measurements. Finally, pediatric populations were not included in this analysis and may cause additional challenges in developing standardized measurement protocols due to the presence of growth plates and bone that has not fully ossified. Our future work aims to further develop 3D FARM to accommodate pediatric populations.

The 3D FARM toolbox enables reproducible and automated extraction of clinically relevant radiographic measurements from WBCT bone models. The demonstrated agreement with manual segmentations and the establishment of adult pathology-specific reference values support its use as a standardized measurement framework for WBCT-based foot and ankle assessment.

## Data Availability

The raw data supporting the conclusions of this article will be made available by the authors, without undue reservation.
