# Supplementary material for: 3D foot and ankle radiographic measurement toolbox
Source: Front Bioeng Biotechnol. 2026 Aug 5;14:1892094. doi: 10.3389/fbioe.2026.1892094 (PMC13486229; doi:10.3389/fbioe.2026.1892094)
Supplement: Supplementary file 1 [file Table1.docx]

**Supplementary Table 1.** Radiographic measurements with group specific intraclass correlation coefficient (ICC) and mean bias.

| **Group** | **Measurement** | **ICC** | **Bias** |
| --- | --- | --- | --- |
| Rectus | C1M | 0.98 | -0.45° |
| Rectus | CIA | 0.97 | -0.05° |
| Rectus | FTP | 0.77 | 0.58% |
| Rectus | HAA 0° | 0.92 | -0.89° |
| Rectus | HAA 20° | 0.92 | -0.82° |
| Rectus | HMA | 0.88 | -1.74 mm |
| Rectus | 1-2IMA | 0.96 | -0.19° |
| Rectus | Trans. Meary’s angle | 0.97 | 0.06° |
| Rectus | Sag. Meary’s angle | 0.92 | 1.27° |
| Rectus | MDTA | 0.87 | -1.26° |
| Rectus | MLCR | 0.81 | 0.00 |
| Rectus | Metatarsal Stacking angle | 0.77 | -0.58° |
| Rectus | Naviculocuboid Overlap | 0.94 | 2.63 |
| Rectus | TTA | 0.82 | 1.14° |
| Rectus | Trans. TCA | 0.95 | -1.24° |
| Rectus | Sag. TCA | 0.91 | -0.82° |
| Rectus | TNA | 0.97 | -0.40° |
| Rectus | TLSA | 0.86 | -0.44° |
| Rectus | Trans. TiCA | 0.94 | 1.07° |
| Rectus | Sag. TiCA | 0.99 | -0.24° |
| Cavus | C1M | 0.99 | -0.85° |
| Cavus | CIA | 0.97 | 0.16° |
| Cavus | FTP | 0.92 | 1.28% |
| Cavus | HAA 0° | 0.98 | -0.80° |
| Cavus | HAA 20° | 0.98 | -0.76° |
| Cavus | HMA | 0.96 | -1.44 mm |
| Cavus | 1-2IMA | 0.87 | 0.04° |
| Cavus | Trans. Meary’s angle | 0.99 | 1.36° |
| Cavus | Sag. Meary’s angle | 0.97 | 1.67° |
| Cavus | MDTA | 0.81 | -1.49° |
| Cavus | MLCR | 0.93 | 0.00 |
| Cavus | Metatarsal Stacking angle | 0.72 | -1.58° |
| Cavus | Naviculocuboid Overlap | 0.97 | 3.80 |
| Cavus | TTA | 0.92 | 1.20° |
| Cavus | Trans. TCA | 0.96 | -0.98° |
| Cavus | Sag. TCA | 0.96 | -0.82° |
| Cavus | TNA | 0.99 | 0.60° |
| Cavus | TLSA | 0.89 | 0.07° |
| Cavus | Trans. TiCA | 0.96 | 0.96° |
| Cavus | Sag. TiCA | 1.00 | -0.12° |
| Planus | C1M | 0.99 | -0.22° |
| Planus | CIA | 0.98 | -0.32° |
| Planus | FTP | 0.84 | 0.36% |
| Planus | HAA 0° | 0.97 | -0.58° |
| Planus | HAA 20° | 0.97 | -0.53° |
| Planus | HMA | 0.96 | -1.35 mm |
| Planus | 1-2IMA | 0.95 | -0.45° |
| Planus | Trans. Meary’s angle | 0.99 | 0.15° |
| Planus | Sag. Meary’s angle | 0.95 | 1.08° |
| Planus | MDTA | 0.82 | -0.92° |
| Planus | MLCR | 0.81 | -0.01 |
| Planus | Metatarsal Stacking angle | 0.72 | -0.34° |
| Planus | Naviculocuboid Overlap | 0.88 | 1.64 |
| Planus | TTA | 0.92 | 1.03° |
| Planus | Trans. TCA | 0.93 | -0.74° |
| Planus | Sag. TCA | 0.91 | -0.86° |
| Planus | TNA | 0.95 | 0.42° |
| Planus | TLSA | 0.94 | -0.45° |
| Planus | Trans. TiCA | 0.98 | 0.76° |
| Planus | Sag. TiCA | 0.99 | -0.45° |
| CMT | C1M | 1.00 | -0.26° |
| CMT | CIA | 0.99 | 0.04° |
| CMT | FTP | 0.99 | 0.07% |
| CMT | HAA 0° | 1.00 | -1.14° |
| CMT | HAA 20° | 1.00 | -1.11° |
| CMT | HMA | 0.99 | -2.17 mm |
| CMT | 1-2IMA | 0.97 | -0.18° |
| CMT | Trans. Meary’s angle | 0.99 | 1.07° |
| CMT | Sag. Meary’s angle | 0.99 | 1.12° |
| CMT | MDTA | 0.35 | -2.11° |
| CMT | MLCR | 0.99 | 0.00 |
| CMT | Metatarsal Stacking angle | 0.99 | 0.13° |
| CMT | Naviculocuboid Overlap | 0.95 | 3.92 |
| CMT | TTA | 0.99 | 1.06° |
| CMT | Trans. TCA | 0.96 | 0.44° |
| CMT | Sag. TCA | 0.84 | -0.86° |
| CMT | TNA | 0.99 | 1.01° |
| CMT | TLSA | 0.59 | -1.62° |
| CMT | Trans. TiCA | 0.95 | 0.01° |
| CMT | Sag. TiCA | 0.99 | 0.35° |
| PCFD | C1M | 0.97 | 0.11° |
| PCFD | CIA | 0.85 | -0.90° |
| PCFD | FTP | 0.96 | -0.01% |
| PCFD | HAA 0° | 0.98 | -1.02° |
| PCFD | HAA 20° | 0.98 | -0.68° |
| PCFD | HMA | 0.98 | -1.14 mm |
| PCFD | 1-2IMA | 0.99 | -0.03° |
| PCFD | Trans. Meary’s angle | 0.98 | -2.03° |
| PCFD | Sag. Meary’s angle | 0.93 | 2.03° |
| PCFD | MDTA | 0.87 | -0.40° |
| PCFD | MLCR | 0.87 | 0.00 |
| PCFD | Metatarsal Stacking angle | 0.90 | 1.05° |
| PCFD | Naviculocuboid Overlap | 0.86 | 1.61 |
| PCFD | TTA | 0.96 | 0.76° |
| PCFD | Trans. TCA | 0.90 | -1.58° |
| PCFD | Sag. TCA | 0.75 | -2.15° |
| PCFD | TNA | 0.97 | -1.78° |
| PCFD | TLSA | 0.92 | -0.66° |
| PCFD | Trans. TiCA | 0.97 | 0.21° |
| PCFD | Sag. TiCA | 0.97 | -0.05° |
| OA | C1M | 1.00 | -0.54° |
| OA | CIA | 0.96 | 0.39° |
| OA | FTP | 0.97 | 0.52% |
| OA | HAA 0° | 0.99 | -0.90° |
| OA | HAA 20° | 0.99 | -0.87° |
| OA | HMA | 0.98 | -1.54 mm |
| OA | 1-2IMA | 0.96 | -0.14° |
| OA | Trans. Meary’s angle | 0.99 | 1.12° |
| OA | Sag. Meary’s angle | 0.99 | 0.68° |
| OA | MDTA | 0.77 | -1.15° |
| OA | MLCR | 0.90 | -0.01 |
| OA | Metatarsal Stacking angle | 0.98 | -0.47° |
| OA | Naviculocuboid Overlap | 0.97 | 2.42 |
| OA | TTA | 0.99 | 0.68° |
| OA | Trans. TCA | 0.96 | -0.74° |
| OA | Sag. TCA | 0.92 | -0.13° |
| OA | TNA | 0.99 | 0.67° |
| OA | TLSA | 0.86 | -0.84° |
| OA | Trans. TiCA | 0.96 | 0.45° |
| OA | Sag. TiCA | 0.98 | -0.20° |

Abbreviations: calcaneal 1st metatarsal angle (C1M), calcaneal inclination angle (CIA), Foot Type Percentage (FTP), hindfoot alignment angle (HAA) at 0° and 20° angles, hindfoot moment arm (HMA), 1st and 2nd intermetatarsal angle (1-2IMA), medial distal tibial angle (MDTA), medial-lateral column ratio (MLCR), talar tilt angle (TTA), talocalcaneal angle (TCA) in the transverse and sagittal planes, talonavicular angle (TNA), tibial lateral surface angle (TLSA), and tibiocalcaneal angle (TiCA) in the transverse and sagittal planes
